# Technical Validation and Clinical Utility of an NGS Targeted Panel to Improve Molecular Characterization of Pediatric Acute Leukemia

**DOI:** 10.3389/fmolb.2022.854098

**Published:** 2022-04-07

**Authors:** Clara Vicente-Garcés, Elena Esperanza-Cebollada, Sara Montesdeoca, Montserrat Torrebadell, Susana Rives, José Luis Dapena, Albert Català, Nuria Conde, Mireia Camós, Nerea Vega-García

**Affiliations:** ^1^ Hematology Laboratory, Hospital Sant Joan de Déu Barcelona, Esplugues de Llobregat, Barcelona, Spain; ^2^ Leukemia and Other Pediatric Hemopathies, Developmental Tumors Biology Group, Institut de Recerca Hospital Sant Joan de Déu, Esplugues de Llobregat, Barcelona, Spain; ^3^ Centro de Investigación Biomédica en Red de Enfermedades Raras (CIBERER), Instituto de Salud Carlos III, Madrid, Spain; ^4^ Pediatric Hematology and Oncology Department, Hospital Sant Joan de Déu Barcelona, University of Barcelona, Barcelona, Spain

**Keywords:** next generation sequencing (NGS), pediatric acute leukemia, clinical impact, molecular diagnostics, precision medicine

## Abstract

Development of next-generation sequencing (NGS) has provided useful genetic information to redefine diagnostic, prognostic, and therapeutic strategies for the management of acute leukemia (AL). However, the application in the clinical setting is still challenging. Our aim was to validate the AmpliSeq™ for Illumina® Childhood Cancer Panel, a pediatric pan-cancer targeted NGS panel that includes the most common genes associated with childhood cancer, and assess its utility in the daily routine of AL diagnostics. In terms of sequencing metrics, the assay reached all the expected values. We obtained a mean read depth greater than 1000×. The panel demonstrated a high sensitivity for DNA (98.5% for variants with 5% variant allele frequency (VAF)) and RNA (94.4%), 100% of specificity and reproducibility for DNA and 89% of reproducibility for RNA. Regarding clinical utility, 49% of mutations and 97% of the fusions identified were demonstrated to have clinical impact. Forty-one percent of mutations refined diagnosis, while 49% of them were considered targetable. Regarding RNA, fusion genes were more clinically impactful in terms of refining diagnostic (97%). Overall, the panel found clinically relevant results in the 43% of patients tested in this cohort. To sum up, we validated a reliable and reproducible method to refine pediatric AL diagnosis, prognosis, and treatment, and demonstrated the feasibility of incorporating a targeted NGS panel into pediatric hematology practice.

## Introduction

Acute leukemia (AL) is the most common pediatric neoplasm and the primary cause of death related to cancer in childhood. It is characterized by the clonal expansion of a myeloid immature progenitor (Acute Myeloid Leukemia, AML) or a lymphoid immature progenitor (Acute Lymphoblastic Leukemia, ALL), with ALL being the most prevalent type of leukemia in children (Hunger and Mullighan, 2015; Grimwade et al., 2016; Bolouri et al., 2017). The survival rates have improved significantly; however, an important proportion of patients still relapse (Gatta et al., 2013). Personalized medicine for the treatment of AL provides a directed therapy for patients based on the comprehensive analysis of different molecular markers that can improve the diagnostic and prognostic algorithms. At the genetic level, pediatric cancers have distinctive features that make them different from adult cancers. Despite also finding gene fusions, copy number variants (CNVs), insertions/deletions (InDels), and epigenetic alterations, pediatric leukemia has a relatively low mutational burden, although generally clinically relevant (Hiemenz et al., 2018). Many current clinical testing of these alterations are laborious, with multiple tests performed separately for a single patient and alteration. In this setting, the development of next-generation sequencing (NGS) techniques has made it possible to address the complexity of AL study (Mullighan, 2013; Izevbaye et al., 2020; Neumann et al., 2754). NGS allows a parallel study of numerous genes and patients with high sensitivity. Although a wide diversity of commercial cancer gene panels is currently available for clinical practice, most of them are focused on adult patients. To supply this lack of pediatric NGS panels, some laboratories have developed NGS custom panels, but this is a very laborious and time-consuming option. Therefore, many laboratories choose to confirm that the commercially available adult-focused NGS panels include also all the relevant genes for the pediatric approximation. Overall, despite the variety of targeted panels to study different types of cancers, the availability of specific panels for pediatric AL is still limited.

The AmpliSeq™ for Illumina® Childhood Cancer Panel is a pediatric pan-cancer NGS targeted panel specific for the study of the most common variants associated with childhood and young adult cancer types. It analyses multiple variant types including gene fusions, hotspot regions, single nucleotide variants (SNVs), InDels, and CNVs in more than one sample at the same time.

This study presents the validation of the AmpliSeq™ for Illumina® Childhood Cancer Panel and its clinical utility in daily routine in AL diagnostics. We assessed its sensitivity, reproducibility, the limit of detection (LOD), and clinical utility. Although this panel analyzes 203 genes involved in different pediatric tumors, this validation is focused on those genes that are relevant for the diagnosis, prognosis, risk stratification, or targeted therapy in pediatric acute leukemia.

## Materials and Methods

### Sample Selection

#### Commercial Controls

Commercial controls were used to assess sensitivity and specificity and to establish the LOD.

As a positive control for the DNA analyses, we used SeraSeq® Tumor Mutation DNA Mix (v2 AF10 HC) (SeraCare, Mildford). This is a multiplex biosynthetic mixture of different clinically relevant DNA variants present at an average variant allele frequency (VAF) of 10%. The genes included are *AKT1, APC, BRAF, CTNNB1, EGFR, ERBB2, FGFR3, FLT3, GNA11, GNAQ, IDH1, JAK2, KIT, KRAS, MPL, NPM1, NRAS, PDGFRA, PIK3CA, PTEN, RET*, and *TP53*.

For the RNA analyses, we used SeraSeq® Myeloid Fusion RNA Mix (SeraCare, Mildford), which is a mixture of synthetic RNA fusions combined with RNA extracted from GM24385 human reference line. We based the studies on *ETV6::ABL1, TCF3::PBX1, BCR::ABL1, RUNX1::RUNX1T1*, and *PML::RARA* fusions.

Negative controls were also needed. We used NA12878 (Coriell Institute of Medical Research) as a DNA negative control and IVS-0035 (Invivoscribe) as an RNA negative control.

#### Patients

We selected 76 pediatric patients diagnosed with B-cell precursors ALL (BCP-ALL) (*n* = 51), T-ALL (*n* = 11), and AML (*n* = 14) from different centers (Hospital Sant Joan de Déu, HSJD; Hospital Clínic de Barcelona, Hospital de la Santa Creu i Sant Pau, Hospital Jerez de la Frontera, Hospital Universitario Ntra. Sra. De Candelaria, Hospital Universitario Miguel Servet, Hospital Universitario Cruces, and Hospital Clínico Universitario Virgen de la Arrixaca) from 2016 to 2020. Out of the 153 patients diagnosed in HSJD during this period, we selected those patients younger than 25 years old with available sample at diagnosis or relapse with high DNA and RNA quality. We also applied a clinical selection criterion, using non-consecutive samples and prioritizing those patients with non-defining genetic results using conventional diagnostic methodologies that could benefit from NGS studies. Consequently, the genetic alterations frequencies from patients included in our study do not reflect the standard distribution of genetic abnormalities in pediatric acute leukemia.

### Molecular Characterization by Conventional Molecular Biology Techniques

The mutational status of *FLT3* (*FLT3* internal tandem duplication, *FLT3-ITD*) and *NPM1* was assessed by labeled-PCR amplification as reported (Gale et al., 2008). *FLT3* tyrosine kinase domain mutations, *cKIT*, and *GATA1* mutations were tested by Sanger sequencing (Kottaridis et al., 2002; Rainis et al., 2003; Manara et al., 2021). The study of the fusion genes *CBFB::MYH11, RUNX1::RUNX1T1, PML::RARA, BCR::ABL1, ETV6::RUNX1, TCF3::PBX1, STIL::TAL1, KMT2A::AFF1 (AF4), KMT2A::MLLT1 (ENL), and KMT2A::MLLT3 (AF9)* was done by quantitative RT-PCR (q-RT-PCR) using specific primers and probes as specified in the Europe Against Cancer Program guidelines (Gabert et al., 2003; Jansen et al., 2021).

### Nucleic Acid Extraction and Quantification

Nucleotide extraction was performed using different methods. DNA was extracted with the Gentra Puregene kit (Qiagen, Hilden, Germany), the QIAamp DNA Mini Kit, or the QIAamp DNA 2.7 Micro Kit (Qiagen, Hilden, Germany). RNA was manually extracted using guanidine thiocyanate-phenol-chloroform method (TriPure, Roche Diagnostics, United States), or using column-based methods with Direct-zol RNA MiniPrep (Zymo Research, California, United States).

The DNA and RNA purity were determined by Quawell Q5000 UV-Vis spectrophotometer (Quawell Technology Inc., San Jose, CA), having all the samples an OD260/280 ratio >1.8. Integrity was assessed by Labchip (PerkinElmer Inc., Courtaboeuf, France), and TapeStation (Agilent, Santa Clara, CA). DNA and RNA concentration were determined by fluorometric quantification using the Qubit 4.0 Fluorimeter (ThermoFisher Scientific, Massachusetts, United States) with the dsDNA BR Assay Kit for DNA samples and the RNA BR Assay Kit for RNA samples.

### AmpliSeq™ for Illumina® Childhood Cancer Panel

Following a PCR-based protocol, the AmpliSeq™ for Illumina® Childhood Cancer Panel analyzes 203 genes per sample simultaneously. It includes 97 gene fusions, 82 DNA variants, 44 full exon coverage, and 24 CNVs.

In our study, we focused on fusion genes, SNVs, and InDels in those genes related to AL. The full list of genes included in the panel is shown in Supplementary Table S1. The list of genes involved in leukemia, and therefore assessed in the validation, are displayed in a different color in the same table.

### Library Preparation and Sequencing

Library preparation was performed using the AmpliSeq™ for Illumina® Childhood Cancer Panel kit (Illumina, San Diego, CA) following the manufacturer’s instructions.

Briefly, a total of 100 ng of DNA was used to generate 3069 amplicons per sample, with an average size of 114 bp, covering coding regions of multiple genes. Simultaneously, 100 ng of RNA per sample was used to study 1701 amplicons, with an average size of 122 bp, targeting gene fusions. RNA was reverse transcribed to cDNA using the Ampliseq^™^ cDNA Synthesis kit (Illumina Inc., San Diego, CA). Amplicon libraries, with specific barcodes for each sample, were generated by performing consecutive PCRs. Quality controls (QC) were done after cleaning up the libraries. Finally, libraries were diluted to 2 nM and then DNA libraries and RNA libraries were pooled at a 5:1 ratio (DNA:RNA). The final pool was diluted to 17–20 p.m. and sequenced on a MiSeq Sequencer with a MiSeq Reagent kit v3 (600 cycles) (Illumina, San Diego, CA). Four patients were sequenced in each run, including paired DNA and RNA.

### Data Analysis

Data obtained from the sequencer was analyzed using the DNA amplicon app and RNA amplicon app (Illumina, Inc.) from BaseSpace^™^ Sequence Hub. Data files were imported to VariantStudio and then raw variants were filtered excluding those with an ExAC population frequency of ≥1%, synonymous variants, and those elsewhere apart from exonic regions. Variants were visualized by using the Integrative Genomics Viewer (IGV) to discard potential artifacts. The remaining variants were manually curated and filtered using various databases including COSMIC (http://cancer.sanger.ac.uk/cosmic), Varsome (https://varsome.com/), and ClinVar (http://www.ncbi.nlm.nih.gov/clinvar), and classified according to the AMP/ASCO/CAP Standards and Guidelines for Somatic Variant Interpretation and Reporting (Li et al., 2017). Turnaround time for this testing was approximately 3 weeks.

### Variant Confirmation

Mutations found by NGS with VAF>15% were confirmed by Sanger sequencing and fusions detected by the panel were confirmed by RT-qPCR, using ABL1 as a housekeeping gene. Different and specific primer sets were designed using PRIMER3plus software (https://primer3plus.com/cgi-bin/dev/primer3plus.cgi). For fusions, 500 ng of total RNA was retrotranscribed with the High Capacity Retrotranscription kit (Applied Biosystems).

To confirm the somatic or germline nature of the variants, we used patient-matched bone marrow samples in complete morphological remission (CR) with negative measurable residual disease (MRD), as assessed by 8-color flow cytometry.

### Analytical Validation

#### Run Metrics

Acceptable sequencing metrics were established by measuring the on-target and uniformity percentages and depth of coverage across the samples sequenced. In accordance with the manufacturer’s instructions, we expected to obtain >95% targets covered at a minimum of 500 × >90% of coverage uniformity, and >80% of on-target aligned reads.

#### Accuracy

Positive and negative commercial controls were used to define true positive (TP), false positive (FP), true negative (TN), and false negative (FN) values. All known variants from positive controls were assigned as TP if they were detected and FN if not. Negative control was a reference sample for different genes. This control enabled us to determine TN when no alteration was detected and FP when there was a variant called. Sensitivity and specificity were calculated from these values. To assess the accuracy, we determined the values of sensitivity [=TP/(TP + FN)) and specificity (=TN/(TN + FP)]. The coefficient of variation (CV) was calculated to evaluate the reproducibility of variant detection by two different persons. We considered acceptable a CV < 20%.

#### Limit of Detection

To determine the LOD, we sequenced different dilutions of the DNA and RNA commercial controls. The SeraSeq® Tumor Mutation DNA Mix was diluted with DNA from the NA12878 negative control to obtain VAFs of 10, 5, 2.5, and 1.25%. For the RNA study, SeraSeq® Myeloid Fusion RNA Mix was diluted with RNA from the IVS-0035 negative control to obtain different dilution ranges (10^−2^, 10^−4^, and 10^−5^).

#### Reproducibility

Reproducibility was assessed by performing the libraries by two different operators and sequencing them in two different runs. This enabled us to quantify the variation introduced by the personnel.

### Determination of Clinical Impact

The clinical impact was determined based on the effect of the biological findings of the panel on diagnosis, prognosis, and potential treatment decision-making. Diagnosis classification was based on the 2016 World Health Organization (WHO) integrated classification of patients. In addition, variants known to contribute to the diagnosis of an AL new subtype were considered based on recent literature. The prognosis for *de novo* ALL patients was assessed following the risk stratification used in the current national treatment protocol SEHOP-PETHEMA 2013. For AML patients, we used the risk stratification of the NOPHO DBH AML 2012 protocol. Those variants defining or contributing to patient risk stratification following new evidence were also considered. Finally, clinical implications regarding treatment were evaluated according to the currently available evidence including the therapeutic protocol, as well as those variants qualifying the patients for an FDA-approved therapy or contributing to be enrolled in a clinical trial. Overall, results were considered clinically useful if they affected and/or contributed to one of these clinically determining factors, corresponding to Tiers 1 and 2 (variants with strong or potential clinical significance) according to the AMP/ASCO/CAP guidelines (Richards et al., 2015). All cases were individually reviewed by at least one hematologist and one molecular expert.

Somatic findings in some genes raised the suspicion of germline origin because the same variant can be both somatic and germline. Variants were considered for further testing if they met the following specific criteria; pathogenic/likely pathogenic variants with VAF > 30%, in genes known to be associated with cancer or if the variant is a known founder mutation or if clinical features suggest a germline origin. In all those cases VAF was carefully interpreted considering purity and ploidy of the sample, germline mosaicism, loss of heterozygosity, CNV in tumor cells, structural rearrangements, and read depth.

### Ethical Issues

The study was conducted in accordance with the ethical standards and the Declaration of Helsinki. All samples were stored in the HSJD Biobank and were used after informed consent was obtained either from the patients and/or from their legal guardians.

## Results

### Analytical Validation

To perform the technical validation of the AmpliSeq™ for Illumina® Childhood Cancer Panel, 80 samples corresponding to 76 patients were sequenced (2 samples were sequenced twice and 2 were the negative and positive commercial controls). Additionally, the 4 commercial controls (2 positive controls from DNA and RNA as well as 2 negative controls from DNA and RNA) were diluted and were sequenced in 2 different runs.

#### Run Metrics

A total of 22 pools were sequenced in a MiSeq sequencer (20 pools containing patients’ samples and 2 pools using different dilutions of commercial samples). All the runs succeeded and completed the sequencing. The failure rate of sequencing for the RNA was 7.7% (7 samples out of 78), while in DNA it was 0%.

Results obtained across the runs revealed an average read per sample of 6.1x10^6^ for DNA. The amplicon mean coverage was 2054× with a coverage uniformity of 97% (range, 70.42–98.82%). A total of 17 amplicons were covered less than 100× and 87 less than 500× from a total of 3069 amplicons. Overall, the percentage of amplicons covered > 500× was 97.2%. The on-target alignment was 96%. All these parameters agreed with the manufacturer’s specifications.

**FIGURE 1:**
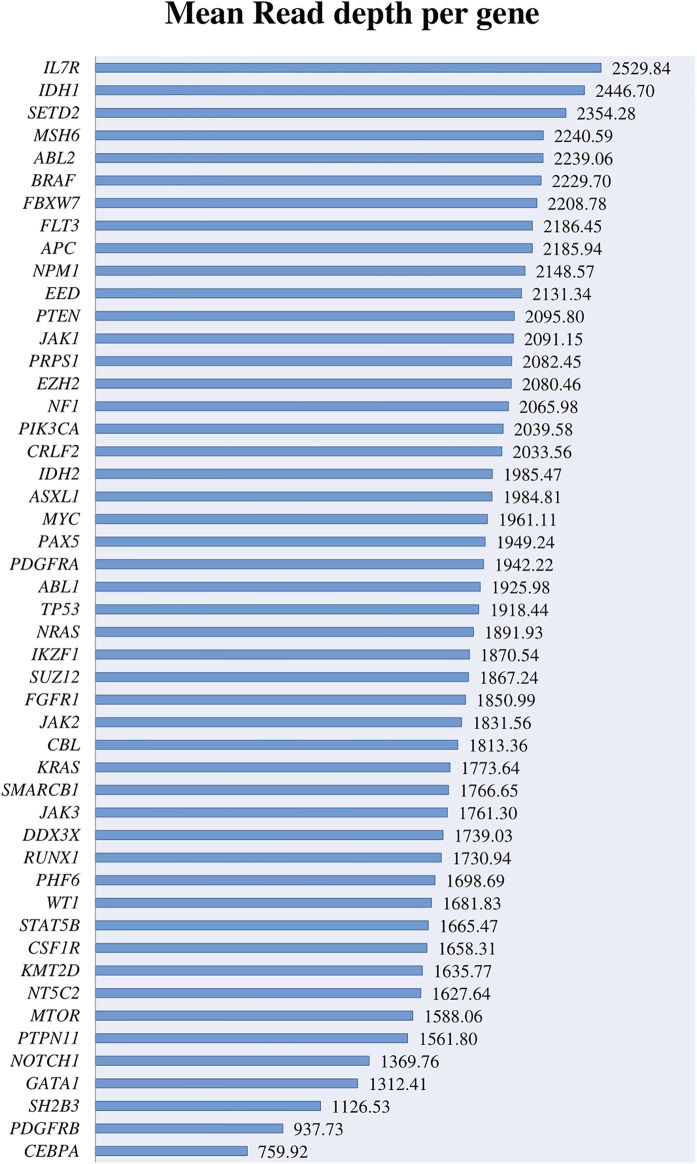
Mean read depth obtained for the different leukemia-related genes.

The most important genes involved in AL and included in the panel were covered above 500× with a depth range from 759.92× to 2529.8× and a mean coverage of 1868.92× (Figure 1). Despite the high coverage of the genes, when we analyzed them in more detail, we found that 28 amplicons covering these genes had a read depth under 500×, and 3 of them < 100× (Supplementary Figures S1A,B). When reviewing these regions in detail, some of them cover regions with low frequent point mutations such as *IKZF1* exon 5, *KMT2D* exons 10, 19, 31, 39, *PTEN* exon 8, *SH2B3* exon 2, and *NOTCH1* exons 26 and 34. However, most of these exons do not represent any known hotspot region for these genes described in the literature.

For the RNA, an average read per sample of 2.5 x 10^6^ with a mean coverage of 1,060x was obtained after sequencing all the samples. The 67.65% (range 50.04–98.34%) of the reads was aligned to the human genome.

#### Accuracy

The accuracy was estimated as the concordance between the variants detected by the AmpliSeq™ for Illumina® Childhood Cancer Panel and the variants reported by the commercial samples’ distributors. The SeraSeq® Tumor Mutation DNA Mix and the NA12878 sample were used to define accuracy in DNA samples. Specificity and sensitivity were calculated at different VAF (10, 5, 2.5, and 1.25%).

**FIGURE 2:**
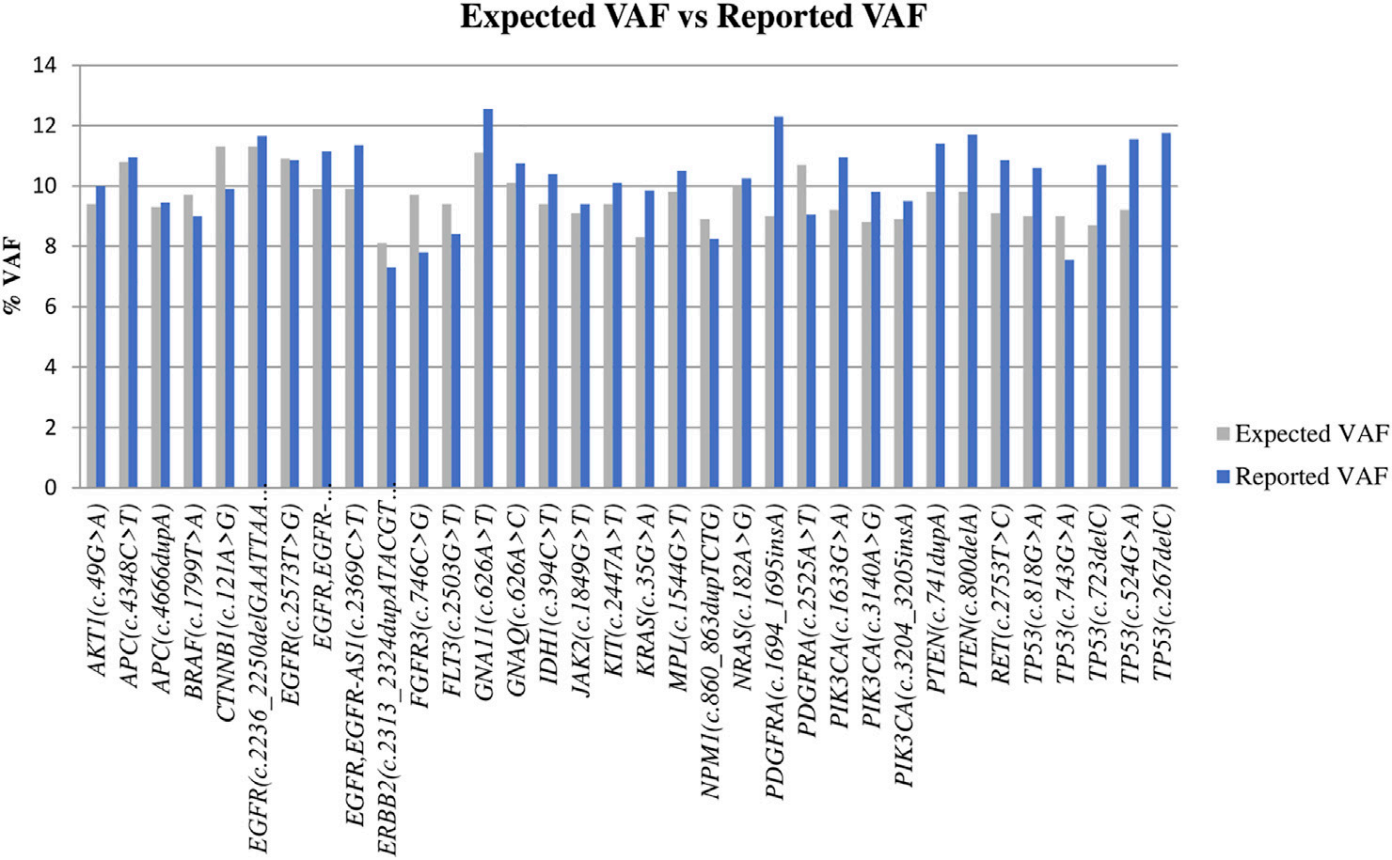
Comparison of the expected VAF and the mean VAF obtained for the different variants. For TP53 (c.267delC), the graphic only shows the value of the reported VAF, as the commercial company did not specify the expected VAF for this variant.

In DNA samples with variants at 10% VAF, all the expected alterations were detected at the expected VAF (Figure 2), and no variants were identified in the known negative samples. Thus, both sensitivity and specificity were 100%. When analyzing samples with a 5% VAF, 1 FN was found but no FP were detected. These results showed 98.5% of sensitivity and 100% of specificity. When we tried to detect variants at 2.5% VAF, the sensitivity decreased to 85.3% and the number of FN exceeded the TP, detecting 18 and 16, respectively. The number of FP was zero, meaning that no variants were found in negative controls and, thus, achieving a specificity of 100%. Finally, at a 1.25% VAF, we were able to detect only 13 positive cases out of 68 TP. In this case, sensitivity drastically decreased to 19.12%, but the specificity remained 100%. Results are summarized in Table 1.

**TABLE 1:** Mean sensitivity across the studied variants obtained for DNA at different VAF and for the undiluted RNA.

	VAF	Sensitivity (%)
DNA	10%	100
	5%	98.5
	2.5%	85.3
	1.25%	19.12
RNA	Undiluted	94.4

To assess the RNA accuracy, the SeraSeq® Myeloid Fusion RNA Mix and the IVS-0035 were used as positive and negative controls, respectively. For fusion detection in RNA samples, all gene rearrangements in SeraSeq® Myeloid Fusion RNA Mix were detected except one (*PML::RARA*), which was not detected by one of the technicians, giving a sensitivity of 94.4% (Table 1). No gene fusions were detected in IVS-0035 RNA negative control. Thus, specificity was 100%.

#### Limit of Detection

The LOD for DNA was assessed for SNVs and small InDels by diluting SeraSeq® Tumor Mutation DNA Mix into NA12878, obtaining different VAFs (10, 5, 2.5, and 1.25%), and then determining the lowest VAF detectable. We were able to detect all the variants at 10% VAF, 98.5% of the variants at a VAF of 5%, 85.3% of the variants at 2.5% VAF, and 19% of the variants at a 1.25% VAF.

To assess the LOD for RNA, the SeraSeq® Myeloid Fusion RNA Mix was diluted into IVS-0035, diluting the initial number of copies to 10^–2^, 10^–4^, and 10^–5^ to determine the lowest number of fusion copies detectable. All the rearrangements were detected on the undiluted sample and the 88.9% (16 out of 18) on the 10^–2^ dilution. When the 10^–4^ and 10^–5^ dilutions were analyzed, no rearrangements were detected (Table 2).

**TABLE 2:** Obtained reads, mean SD, and %CV for undiluted RNA and 10^–2^ dilution for each of the fusion genes analyzed. Libraries were performed by two operators (A and B).

Gene Id	Hgvs	Operator A		Operator B		Mean		SD		% CV	
		Undiluted RNA reads	10^−2^ dilution reads	Undiluted RNA reads	10^−2^ dilution reads	Undiluted RNA reads	10^−2^ dilution reads	Undiluted RNA reads	10^−2^ dilution reads	Undiluted RNA reads	10^−2^ dilution reads
*BCR::ABL1*	BCR(NM_004327.3):r.1_3378 ABL (NM_005157.3):r.83_5384	40,916	2014	40,376	1168	40,646	1,591	381.8	598.2	1	38
*ETV6::ABL1* (*transcript 1*)	ETV6(NM_001987.4):r.1_737 ABL1(NM_007313.2):r.576-5881	40,128	672	15,024	678	27,576	675	17,751.2	4.2	64	1
*ETV6::ABL1* (*transcript 2*)	ETV6(NM_001987.4):r.1_1283 ABL1(NM_007313.2):r.576-5881	15,728	1052	20,890	676	18,309	864	3,650.1	265.9	20	31
*FIP1L1::*>*PDGFRA*	FIP1L1(NM_030917.3):r.1_1109 PDGFRA(NM_006206.5):r.2037_6590	54,960	4104	44,730	1938	49,845	3021	7,233.7	1,531.6	15	51
*MYST3::*>*CREBBP*	MYST3(NM_006766.4):r.1_3803 CREBBP(NM_004380.2):r.290_10197	20,532	1066	26,814	680	23,673	873	4,442.0	272.9	19	31
*PCM1::JAK2*	PCM1(NM_006197.3):r.1_4365 JAK2(NM_004972.3):r.2008_5285	17,866	974	26,152	736	22,009	855	5,859.1	168.3	27	20
*PML::RARA*	PML (NM_033238.2):r.1_1786_ ins134bp RARA (NM_000964.3):r.657_3,301	Not detected	Not detected	9,396	Not detected	9,396^†^	—	—	—	—	—
*RUNX1::*>*RUNX1T1*	RUNX1 (NM_001754.4): r.1-803 RUNX1T1 (NM_004349.3):r.419-7420	11,028	856	12,968	462	11,998	659	1,371.8	278.6	11	42
*TCF3::PBX1*	TCF3(NM_003200.3):r.1_1519 PBX1(NM_002585.3):r.729_6918	21,434	1842	23,426	868	22,430	1,355	1,408.6	688.7	6	51

SD: standard deviation; %CV: percent coefficient of variation.

^†^ This value is not a mean as it was detected by just one operator.

#### Reproducibility

The concordance of the different variants called between the runs performed by different laboratory technicians was used to decide whether the performance of the libraries was reproducible.

**FIGURE 3:**
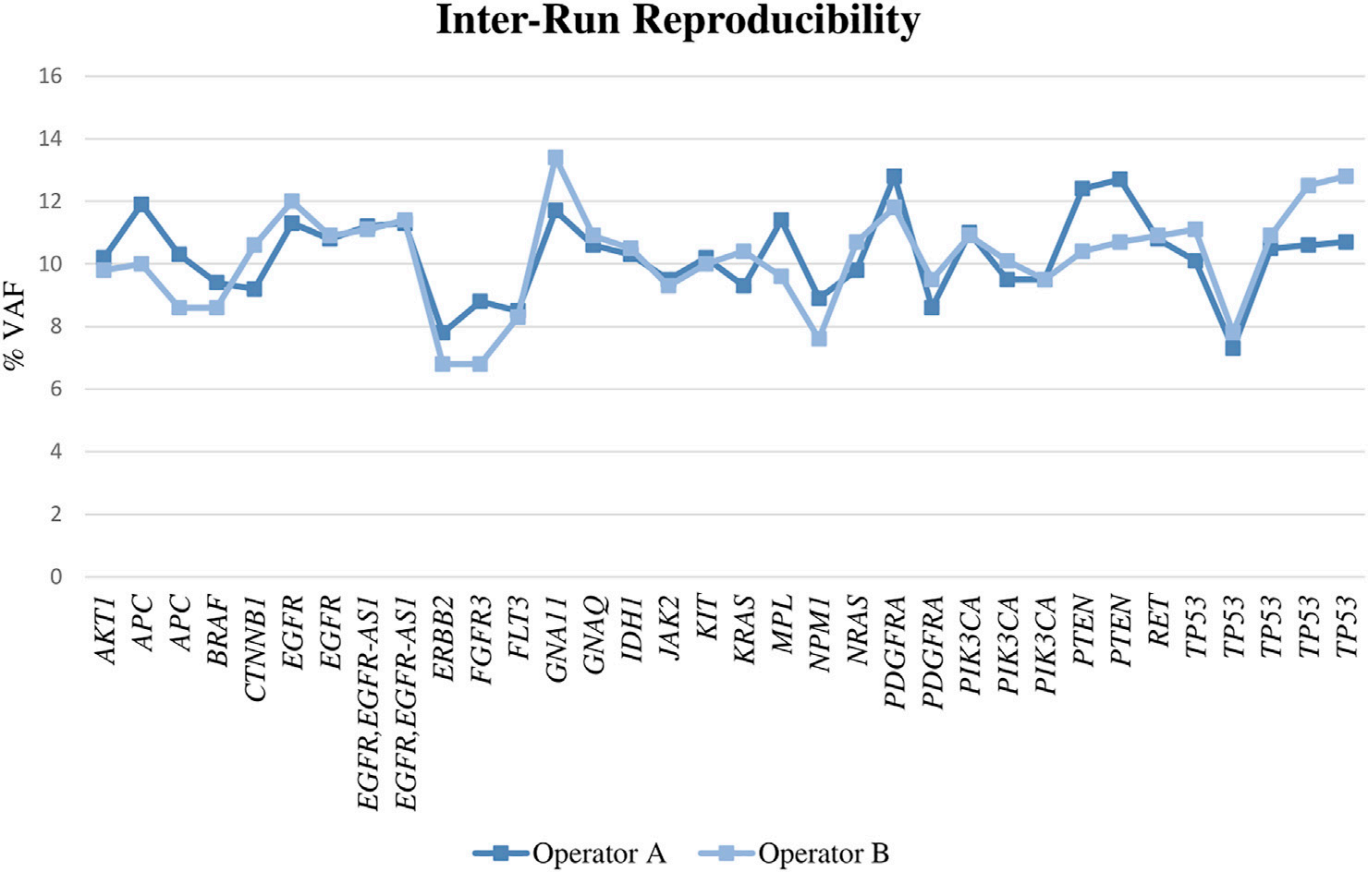
VAF obtained for the assessed genes performing the libraries by two different operators.

For DNA, we found a reproducibility of 100%, as all the alterations were called in both runs with a similar VAF (Figure 3; Supplementary Table S2) and the CV was fewer than 20% in all cases (range, 0–18% with a mean of 6%). When the samples were diluted, the results obtained by both operators were also reproducible in most of the cases.

Regarding the RNA, to analyze the reproducibility we examined the same samples on two different runs performed by two different operators. Reproducibility was 89% for the rearrangements tested, as Operator 1 was not able to detect one of the fusion genes (Table 2).

### Clinical Performance

#### Patients’ Characteristics

After completing the technical validation study with commercial controls, the clinical utility of the AmpliSeq™ for Illumina® Childhood Cancer Panel was evaluated using non-consecutive clinical samples, priming the cohort in patients with unknown genetics and patients with not-stratifying genetics according to conventional methodologies.

In total, 76 patients were included in the study. The median age was 6.8 years (range, 0.22–25 years). Thirty-eight (50%) were men and 38 (50%) were women. Overall, 62 corresponded to ALL patients (51 BCP-ALL and 11 T-ALL) and 14 AML.

#### Mutation Distribution

**FIGURE 4:**
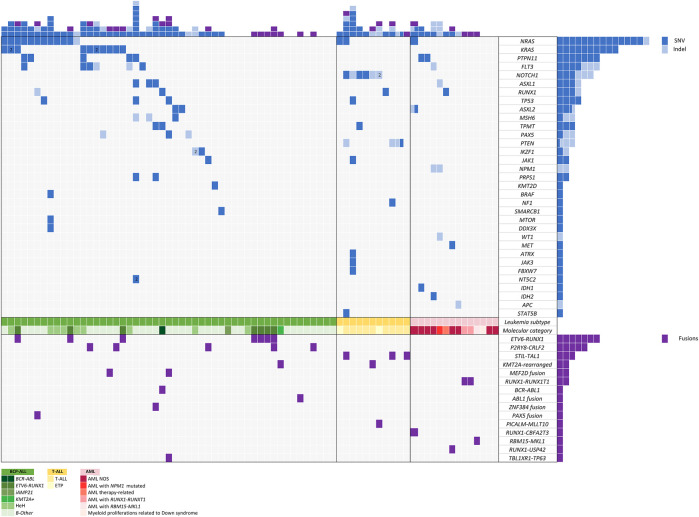
Mutation and gene fusion distribution across the different patients analyzed.

After variant prioritization, 99 variants in 33 genes were further considered as relevant in the 76 samples. The mean number of mutations per sample was 1.3 (range, 1 to 7 mutations per sample). Thus, 68% of patients (*n* = 52) showed at least one mutation. No mutations were detected in 24 patients. All variants are detailed in Supplementary Table S3, and their distribution per gene and patient is shown in Figure 4.

Seventy-eight percent of variants (*n* = 77) were SNVs and 22% (*n* = 22) were InDels with a mean allele frequency of 41.73% (range, 5–87.7%). Among the 203 genes of the DNA panel, 52 genes were selected considering those previously reported as being involved in pediatric acute leukemia. Among these, 33 genes were mutated in our study: *NRAS, KRAS, PTPN11, FLT3, NOTCH1, ASXL1, RUNX1, TP53, ASXL2, MSH6, TPMT, PAX5, PTEN, IKZF1, JAK1, NPM1, PRPS1, KMT2D, BRAF, NF1, SMARCB1, MTOR, DDX3X, WT1, MET, ATRX, JAK3, FBXW7, NT5C2, IDH1, IDH2, APC,* and *STAT5B*.

A high rate of variants in genes involved in signal transduction and driver genes were observed. The most frequently identified variants were in *NRAS* (*n* = 14), *KRAS* (*n* = 10), *PTPN11* (*n* = 7), *FLT3* (*n* = 6), *NOTCH1* (*n* = 6), *ASXL1* (*n* = 3), *RUNX1* (*n* = 4), and *TP53* (*n* = 3), all of them involving hotspot regions. Mutations in genes involved in signaling pathways were the most frequent and generally showed the lowest median VAF (5–30%) (*NRAS, KRAS, PTPN11*, and *FLT3*).

#### Fusion Distribution

We observed 15 different fusions in 30 of 76 patients. Fifteen patients harbored well-known frequent recurrent fusions currently employed to stratify patients in therapeutic protocols, including *ETV6::RUNX1, BCR::ABL1, KMT2A*-rearrangements, and *RUNX1::RUNX1T1*. The remaining patients harbored already reported fusions that do not impact our current treatment protocols (*STIL::TAL1, RBM15::MKL1, PICALM::MLLT10*) and novel fusions recently reported in the literature with potential or clinical impact in therapeutic protocols [*P2RY8::CRLF2, MEF2D::BCL9, MEF2D::CSF1R, ETV6::ABL1, TCF3::ZNF384, PAX5::NOL4L, RUNX1::CBFA2T3, RUNX1::USP42*, and *TBL1XR1::TP63* (Figure 4)]. All these fusions were considered clinically significant and were confirmed by RT-qPCR. The most frequent fusion was *ETV6::RUNX1* (*n* = 7), followed by *P2RY8::CRLF2* (*n* = 5) and *STIL::TAL1* (*n* = 3).

Overall, 30 of 76 patients (39%) were found to carry fusions with potential clinical impact.

#### Clinical Impact of Genomic Findings

As mentioned, compared to the conventional methodologies, the AmpliSeq™ for Illumina® Childhood Cancer Panel allows us to analyze more genes or hotspots in a single assay.

A total of 99 variants were classified as pathogenic variants. Altogether, based on current AL clinical approved guidelines and recently published studies, 49% (*n* = 48/99) of the variants were clinically relevant. Furthermore, the variants were classified according to their capacity to establish or refine the patient’s prognosis and to identify associated targeted therapies. Based on current protocols and recent literature, 70% of the variants could be potentially used for pediatric acute leukemia risk stratification and 41% for treatment selection.

Fusions were present in 39% of patients (30/76), and all patients but one carried clinically significant fusions based on currently published studies. Only one patient had a fusion of uncertain clinical significance (*TBLX1::TP63*). Among the 29 patients harboring clinically significant fusions, the genomic finding helped to refine the diagnosis or prognosis, and in 10 cases (10/29, 34%) provided evidence for targeted therapy.

**FIGURE 5:**
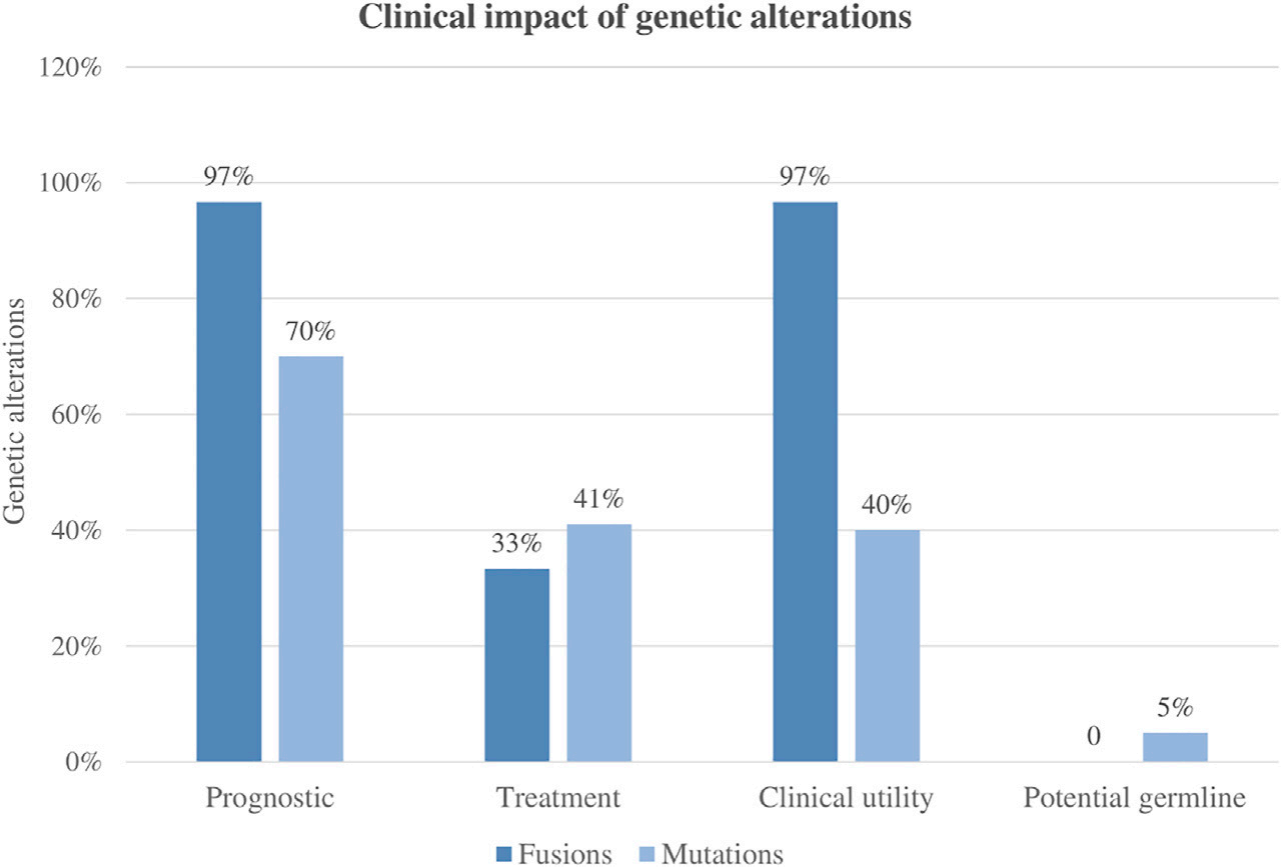
Clinical impact of panel sequencing regarding different genetic alterations.

Overall, the clinical impact following international guidelines was 97% (29/30), meaning that most of the patients enrolled in the NGS studies could benefit from the genomic findings derived from the AmpliSeq™ for Illumina® Childhood Cancer Panel analysis. Of note, in 38% of patients (11/29), these molecular markers would be missed by conventional molecular techniques (Figure 5).

**FIGURE 6:**
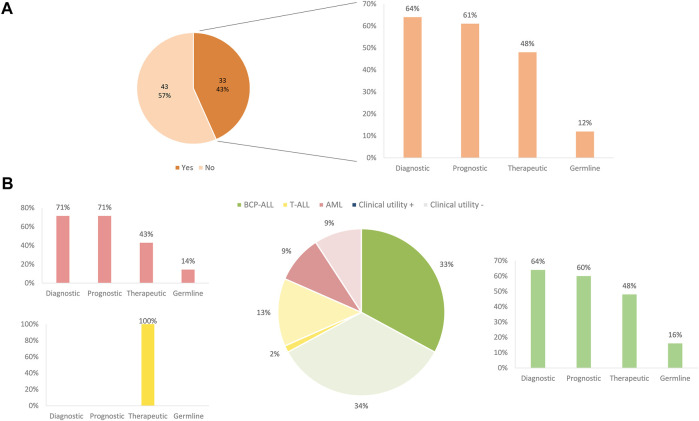
Clinical impact of panel sequencing by subtype of leukemia. **(A)** Overall clinical impact classified by the benefit apported. **(B)** Clinical impact and benefit category depending on the different subtype of leukemia.

Following a case review of each patient tested and the overall clinical impact of comprehensive results for a given patient, 43% of all patients derived a refined diagnostic, prognostic, and/or therapeutic benefit from comprehensive genomic testing. In BCP-ALL and AML, testing with the AmpliSeq™ for Illumina® Childhood Cancer Panel, most often impacted patients by refining the diagnosis (64%). Moreover, genomic results were also particularly impactful for prognosis in 61% of these patients and therapeutic impact, evaluated in terms of information for potential targeted therapy, was identified in 48% of patients. In addition, 6% of patients, including both BCP-ALL and AML, showed variants with suspected germline variants (Figure 6).

Globally, considering DNA mutations and fusion genes, 83% of patients (63/76) showed at least one genetic alteration, and the NGS panel revealed additional molecular markers that could be used for pediatric AL classification, prognosis, or treatment selection. No genetic alteration was detected in 13 patients (cytogenetics of these patients is available in Supplementary Table S4). Finally, alterations in genes *IKZF1, MSH6, RUNX1, TP53,* and *TPMT* raised suspicion of germline predisposition in 13 patients, all of whom were referred to the cancer predisposition program for genetic counseling and confirmatory germline testing. However, we only confirmed a germline origin in genes *MSH6, TP53,* and *TPMT* in 5 patients (6%).

## Discussion

The development of NGS in clinical laboratories has allowed a better characterization of leukemia leading to a better clinical management of the patients. However, the constant evolution of this methodology, the wide range of panels and platforms, and the lack of established universal standard quality criteria for NGS, its application to a routine diagnostics laboratory needs to be individually validated (Jennings et al., 2017).

Here we describe the validation of a pediatric NGS cancer panel, AmpliSeq™ for Illumina® Childhood Cancer Panel, as well as its clinical utility, in a cohort of 76 patients diagnosed with AL.

### Analytical Validation

The quality criteria established in the present study was based on panel manufacturers’ expected yield, previous reports from clinical laboratories that validated other NGS panels, and guidelines for NGS implementation in diagnostics (Richards et al., 2015; De Leng et al., 2016; Jennings et al., 2017; Li et al., 2017; Hirsch et al., 2018). In terms of sequencing metrics (mean read depth, uniformity, and so forth) the AmpliSeq™ for Illumina® Childhood Cancer Panel performance in our laboratory reached all the expected values and similar results to those obtained in other panels or designs sequenced on Illumina platforms (Vega-Garcia et al., 2020). The assessment of the mean read depth per amplicon showed good quality in most of the analyzed fragments. The mean read depth obtained overcame the expected, as we reached a mean greater than 1000×. These results technically validate the NGS assay and grant the identification of variant detection at high depth, which is important when analyzing somatic tumor samples with variants at low VAF (Richards et al., 2015) and reaching a high level of sensitivity and specificity (see below). In our study, there were some regions from 5 different genes (*IKZF1, NOTCH1, KMT2D, PTEN,* and *SH2B3*) where we did not obtain the expected read depth. Thus, when performing this NGS panel in clinical practice, it will be necessary to check those regions using alternative techniques such as Sanger sequencing to ensure that there are no missed variants. Additionally, before implementing any NGS panel, it is necessary to confirm that it includes all the genetic relevant variants for our disease of interest. The AmpliSeq™ for Illumina® Childhood Cancer Panel covers most of the common variants observed in leukemia patients, as well as recently defined genetic subtypes. However, some rare or novel variants are lacking. One alternative to overcome this problem could be to customize the panel adding the absent genes, making the panel complete and adapted to our disease.

The AmpliSeq™ for Illumina® Childhood Cancer Panel demonstrated good sensitivity and specificity for both mutations and gene fusions. We achieved a sensitivity of 100% for variants with a 10% VAF and a sensitivity of 98.5% for variants with 5% VAF. Moreover, for RNA, the sensitivity achieved was 94.4%, and the specificity reached 100%. When filtering, the number of false positive variants can be reduced, increasing the specificity, by performing a visual inspection of these variants using tools such as IGV. Although an LOD of 2.5% VAF for DNA was achieved in 85.3% of the cases, following international guidelines (Li et al., 2017) the cutoff threshold for variant reporting was set at 5%, in which we were able to detect 95% of the samples. Thus, the implementation of NGS allowed us the detection of variants with low VAF and clonal heterogeneity, a common feature in AL (Swaminathan et al., 2015; Ferrando and López-Otín, 2017; Oshima et al., 2019).

Finally, we demonstrated reproducibility of almost 100% with a CV fewer than 20% regarding DNA; for the RNA we obtained 89% of reproducibility. Altogether, our data showed that, regardless of the technician processing the libraries, we were able to detect the same variants in most of the cases. Thus, it was not a hand-dependent finding, which is important when implementing a new methodology in a routine clinical laboratory.

The number of variants per case was relatively low, reflecting the low tumor mutational burden (TMB) observed in pediatric tumors in contrast to adult tumors, except for those cases containing pathogenic variants in mismatched repair genes (Vogelstein et al., 2013; Gröbner et al., 2018). The general landscape of mutations observed in our cohort matches data observed from recent large-scale pediatric leukemia studies. Somatic mutation and fusion distribution reflecting patterns of co-occurrence and exclusivities and the suspected presence of germline predisposition were similar to other studies (Mullighan et al., 2007; Inaba and Mullighan, 2020; Conneely and Stevens, 2021; Klco and Mullighan, 2021; Quessada et al., 2021; Surrey et al., 2021). In particular, the most significantly common mutated genes that these studies identified were driver leukemogenic genes and genes involved in signaling pathways, which were also highly recurrent in our cohort (Holmfeldt et al., 2013; Mullighan, 2013; Lindqvist et al., 2015; Paulsson et al., 2015; Tran and Hunger, 2020; Wang et al., 2020). The VAF analysis of the variants per gene agreed with recent publications, suggesting that mutations in specific driver genes (*NOTCH1, ASXL1, NPM1*) can be associated with clonal hematopoiesis. These mutations are suggested to be acquired by preleukemic cells and can be stable for years or, alternatively, can be acquired during leukemogenesis and be present in the founding clone (Young et al., 2016; Ferrando and López-Otín, 2017). On the other hand, genes in RAS-pathway tend to be subclonal (Jerchel et al., 2018). In this regard, the sensitivity of the NGS compared with conventional techniques is especially important because patients harboring these subclonal mutations could benefit from targeted therapy.

In the same line, some of the most recurrent fusion genes in pediatric leukemia were found in our cohort (*ETV6::RUNX1, STIL::TAL1, KMT2A*-rearrangements, *RUNX1::RUNX1T1, BCR::ABL1*), as well as other novel rearrangements described, most of them associated with BCP-ALL B-other subtypes (*MEF2D::BCL9, MEF2D::CSF1R, ETV6::ABL1, TCF3::ZNF384,* and *PAX5::NOL4L*) or to rare and/or recent AML fusions reported in the literature (*RUNX1::CBFA2T3, RBM15::MKL1, RUNX1::USP42,* and *TBL1XR1::TP63*) (Medinger and Passweg, 2017; Schwab and Harrison, 2018; Inaba and Mullighan, 2020). Of note, the performing for PML:RARA fusion was not reproducible, as only one technician could detect this fusion. Regarding this, authors reviewed the raw RNA data to look for the fusion and were able to find it: it was probably missed by some parameters applied by Illumina RNA Amplicon algorithm analysis. This highlights the need to validate the techniques in the laboratory before using them to identify their weaknesses and, importantly, to integrate the molecular results in the clinical settings. In this particular case, if the initial clinical, morphological, and phenotypical features raise the suspicion of acute promyelocytic leukemia, the emergency of the situation and the fast diagnosis needed to discard the PML:RARA fusion would favor the use of the other methodologies in the first place.

Despite the difficulty to incorporate all the relevant molecular abnormalities described and remain updated, targeted panels are easy to integrate into clinical laboratories. In this regard, one of the main pitfalls of this panel is the absence of some important genes such as *NUTD15*, *MTHFR*, and *CEP72*, as they are related to drug metabolism and response in new therapeutic protocols in ALL (Maamari et al., 2020). Moreover, some interesting rearrangements such as *MNX1::ETV6, DUX4*-, *ERG*-rearrangements, and *IGH* rearrangements (Inaba and Mullighan, 2020; Quessada et al., 2021) are missed.

### Clinical Impact

Regarding the clinical utility of the panel, overall, 49% of mutations and 97% of the identified fusions were demonstrated to have a clinical impact by recent publications and new proposals for pediatric treatment (Inaba and Mullighan, 2020; JS and SE M, 2021). Regarding mutations, approximately 41% of them refined the diagnosis, while 49% were considered targetable. On the contrary, fusion genes were more clinically impactful in terms of refining diagnosis (97%) than for applying targeted therapies. In this sense, it is important to consider the heterogenic complexity of overlapping findings within patients.

Considering the number of patients who benefited from the targeted-NGS panel, our results showed that the results obtained by the AmpliSeq™ for Illumina® Childhood Cancer Panel were clinically relevant in 43% of the patients tested in this cohort. This rate of actionable alterations is similar to that detected in other published clinical sequencing studies in pediatric oncology, which showed between 30 and 60% rate of potentially targetable alterations and around 10% of germline mutations (Alonso et al., 2019; Ishida et al., 2019; Oberg et al., 2021; Surrey et al., 2021). Nevertheless, targeted panels contain a restricted number of genes, usually the most mutated, which may make difficult the identification of rare or novel variants, as some of them could be missed. To have a wider vision of the genetic landscape of the patients, it would be necessary to perform wider NGS strategies such as whole exome sequencing (WES) or whole genome sequencing (WGS). However, these approaches require more hands-on time, are more complex, and produce a huge amount of information, which translates into a longer analysis time and an unacceptable turnaround time to response in clinical practice. Therefore, choosing a targeted NGS panel adapted to our disease is a good option, which can be combined with other conventional methodologies that provide us complementary information for the diagnosis.

Panel testing in our cohort had an impact on refining diagnosis and prognosis in approximately 60% of the patients, especially in AML and BCP-ALL. However, if only those patients with no genetic diagnosis by conventional methods were included, the expected clinical impact of the panel would be higher. The increasing knowledge on the genetic landscape of pediatric AL allows the proposal of new classifications based on molecular alterations that also can be used as markers for targeted therapy or follow-up of drug response based on MRD. In fact, new categories based on new rearrangements and mutational status have been added to the 2016 World Health Organization classification (Arber et al., 2016; Wang and He, 2016). In this regard, the panel design can detect most of these novel mutations and rearrangements. Although some of the information gained from this panel could also be obtained through other means (karyotyping, FISH, or qPCR), conventional approaches may be insufficient to stratify patients into recent proposals (Carbonell et al., 2019; Coccaro et al., 2019). Thus, the AmpliSeq™ for Illumina® Childhood Cancer Panel provides comprehensive molecular analysis with increased efficiency avoiding the need for staged molecular testing. Despite this, the need of combining different diagnostic approaches including NGS must be carefully evaluated by each laboratory, especially to confirm/discard those alterations that may modify the clinical management.

Targeted NGS panels, such as AmpliSeqTM for Illumina® Childhood Cancer Panel, provide us with information of hundreds of genes in a brief time, which is essential in clinical practice. Depending on the local resources, the number of cases, and the urgency to apply the results, it allows us to adapt the number of patients to be analyzed or the machine to run the libraries. In general, the turnaround time for AmpliSeqTM for Illumina® Childhood Cancer Panel is approximately 7 d for 4 patients in a MiSeq system. However, it could be reduced to 3–4 d analyzing only one sample in a MiniSeq system. The panel allows us to adapt to a local setting and plan the application of a combination of conventional and NGS approaches in a stepwise manner. Now, there are few FDA-approved targeted leukemia therapies available to pediatric patients, yet there are novel leukemia subtypes in which targeted therapy (FDA-approved and off-label) is accepted as best practice in the upfront or relapse setting. This includes the use of imatinib or dasatinib in ABL-class fusions patients or ruxolitinib for *JAK2*-mutant patients (Roberts et al., 2014; Pui, 2020; Kantarjian et al., 2021). In our cohort, approximately 50% of patients would potentially have a targetable alteration supported by an FDA-approved drug or experimental drug with preclinical evidence (approved with other indication or age). Furthermore, despite it was out of the scope of this study, germline variant testing in *TPMT* and *NUTD15* is essential given that certain polymorphisms in these genes lead to altered metabolism of the therapeutic agents and can inform about the requirement of adjusting the dosage of thioguanine and mercaptopurine (McLeod et al., 2000; Maxwell and Cole, 2017; Singh et al., 2017). In this regard, the AmpliSeq™ for Illumina® Childhood Cancer Panel only allowed the identification of *TPMT* variants.

Somatic sequencing can also identify potential underlying germline variants and subsequent cancer predisposition syndromes, as some of the genes involved are the same for leukemia patients (Obrochta and Godley, 2018; Bloom et al., 2020; Klco and Mullighan, 2021). Both ALL and AML patients had a rate of around 10% of possible germline alterations requiring additional confirmation and possible follow-up with the cancer predisposition unit. In our cohort, the somatic mutations confirmed to be of germline origin, by testing them in an MRD-negative remission sample were in *TP53* and *MSH6* genes, in line with the previous reports (Kraft and Godley, 2020; Klco and Mullighan, 2021). However, it is important to keep in mind that this panel is not designed for this purpose, missing some important genes related to cancer predisposition (Desai et al., 2017).

In the aggregate, sequencing results provide extra valuable information clinically relevant for diagnostic, prognostic, and pharmacogenomics purposes, such as resistance alleles and subclones, as well as information related to clonal evolution in the case of study-matched samples, where all of them do not meet the definition of “actionability”.

## Conclusion

Our study shows that targeted NGS-based assay is reproducible and precise; therefore, it is applicable in the pediatric AL diagnostic workup after careful validation. Thus, the AmpliSeq™ for Illumina® Childhood Cancer Panel testing can be efficiently incorporated into clinical care, providing useful molecular information for diagnosis, prognosis, treatment, and follow-up. In our selected cohort of patients, the comprehensive panel testing had a meaningful impact on clinical care, including diagnosis, prognosis, and treatment planning. Moreover, with the increase in precision medicine programs and the knowledge acquired in integrating all the molecular data, the adoption of a more inclusive definition of clinical impact would increase the benefits of incorporating NGS technologies in clinical practice.

## Data Availability

The original contributions presented in the study are included in the article/supplementary material, further inquiries can be directed to the corresponding author.
