# Supplementary material for: Technical Validation and Clinical Utility of an NGS Targeted Panel to Improve Molecular Characterization of Pediatric Acute Leukemia
Source: Front Mol Biosci. 2022 Apr 7;9:854098. doi: 10.3389/fmolb.2022.854098 (PMC9021638; doi:10.3389/fmolb.2022.854098)
Supplement: Supplementary file 2 [file DataSheet1.docx]

**Supplementary table 1.** AmpliSeq^TM^ for Illumina® Childhood Cancer Panel gene content for DNA and RNA. Genes known to be involved in leukemia are highlighted in orange.

| **DNA** | | | | | | | | **RNA** | | | | |
| --- | --- | --- | --- | --- | --- | --- | --- | --- | --- | --- | --- | --- |
| **HotSpot** | | | | | **Full exon coverage** | | | **Fusion** | | | | |
| ABL1 | CSF3R | GATA2 | MAP2K1 | PIK3R1 | APC | GATA3 | RUNX1 | ABL1 | FGFR2 | MEF2D | NTRK3 | ROS1 |
| ABL2 | CTNNB1 | GNA11 | MAP2K2 | PPM1D | ARID1A | GNA13 | SMARCA4 | ABL2 | FGFR3 | MET | NUP214 | RUNX1 |
| ALK | DAXX | GNAQ | MET | PTPN11 | ARID1B | ID3 | SMARCB1 | AFF3 | FLT3 | MKL1 | NUP98 | SS18 |
| ACVR1 | DNMT3A | H3F3A | MPL | RAF1 | ATRX | IKZF1 | SOCS2 | ALK | FOSB | MLLT10 | NUTM1 | SSBP2 |
| AKT1 | EGFR | HDAC9 | MSH6 | RET | CDKN2A | KDM6A | SUFU | BCL11B | FUS | MN1 | NUTM2B | STAG2 |
| ASXL1 | EP300 | HIST1H3B | MTOR | RHOA | CDKN2B | KMT2D | SUZ12 | BCOR | GLI1 | MYB | PAX3 | STAT6 |
| ASXL2 | ERBB2 | HRAS | MYC | SETBP1 | CEBPA | MYOD1 | TCF3 | BCR | GLIS2 | MYBL1 | PAX5 | TAL1 |
| BRAF | ERBB3 | IDH1 | MYCN | SETD2 | CHD7 | NF1 | TET2 | BRAF | HMGA2 | MYH11 | PAX7 | TCF3 |
| CALR | ERBB4 | IDH2 | NCOR2 | SH2B3 | CRLF1 | NF2 | TP53 | CAMTA1 | JAK2 | MYH9 | PDGFB | TFE3 |
| CBL | ESR1 | IL7R | NOTCH1 | SH2D1A | DDX3X | PHF6 | TSC1 | CCND1 | KAT6A | NCOA2 | PDGFRA | TP63 |
| CCND1 | EZH2 | JAK1 | NPM1 | SMO | DICER1 | PRPS1 | TSC2 | CIC | KMT2A | NCOR1 | PDGFRB | TSLP |
| CCND3 | FASLG | JAK2 | NRAS | STAT3 | EBF1 | PSMB5 | WHSC1 | CREBBP | KMT2B | NOTCH1 | PLAG1 | TSPAN4 |
| CCR5 | FBXW7 | JAK3 | NT5C2 | STAT5B | EED | PTCH1 | WT1 | CRLF2 | KMT2C | NOTCH2 | RAF1 | UBTF |
| CDK4 | FGFR1 | KDM4C | PAX5 | TERT | FAS | PTEN | XIAP | CSF1R | KMT2D | NOTCH4 | RANBP17 | USP6 |
| CIC | FGFR2 | KDR | PDGFRA | TPMT | GATA1 | RB1 |  | DUSP22 | LMO2 | NPM1 | RARA | WHSC1 |
| CREBBP | FGFR3 | KIT | PDGFRB | USP7 |  |  |  | EGFR | MAML2 | NR4A3 | RECK | YAP1 |
| CRLF2 | FLT3 | KRAS | PIK3CA | ZMYM3 |  |  |  | ETV6 | MAN2B1 | NTRK1 | RELA | ZMYND11 |
| CSF1R |  |  |  |  |  |  |  | EWSR1 | MECOM | NTRK2 | RET | ZNF384 |
|  |  |  |  |  |  |  |  | FGFR1 |  |  |  |  |

|  |  |  |  | **Operator A** | | | | **Operator B** | | | | **Mean** | | | | **SD** | | | | **CV%** | | | |
| --- | --- | --- | --- | --- | --- | --- | --- | --- | --- | --- | --- | --- | --- | --- | --- | --- | --- | --- | --- | --- | --- | --- | --- |
| **GEN ID** | **TYPE MUTATION** | **HGVS NOMENCLATURE** | **VAF**  **expected** | **VAF 10**  **%** | **VAF 5**  **%** | **VAF 2.5%** | **VAF 1.25%** | **VAF 10**  **%** | **VAF 5**  **%** | **VAF 2.5%** | **VAF 1.25%** | **VAF 10**  **%** | **VAF 5**  **%** | **VAF 2.5%** | **VAF 1.25%** | **VAF 10**  **%** | **VAF5  %** | **VAF 2.5%** | **VAF 1.25%** | **VAF 10**  **%** | **VAF5  %** | **VAF 2.5%** | **VAF 1.25%** |
| ***AKT1*** | snv | c.49G>A | 9.4 | 10.2 | 4.5 | 2.4 | - | 9.8 | 3.3 | 2.3 | - | 10 | 3.9 | 2.35 | - | 0.28 | 0.85 | 0.07 | - | 3 | 22 | 3 | - |
| ***APC*** | snv | c.4348C>T | 10.8 | 11.9 | 3.9 | 2.8 | 1.5 | 10 | 4.6 | 1.9 | - | 10.9 | 4.3 | 2.35 | 1.5† | 1.34 | 0.49 | 0.64 | - | 12 | 12 | 27 | - |
| ***APC*** | insertion | c.4666dupA | 9.3 | 10.3 | 3.1 | 2.4 | - | 8.6 | 4.4 | - | - | 9.5 | 3.75 | 2.4† | - | 1.20 | 0.92 | - | - | 13 | 25 | - | - |
| ***BRAF*** | snv | c.1799T>A | 9.7 | 9.4 | 3.8 | 2.3 | - | 8.6 | 3.6 | 2.2 | - | 9 | 3.7 | 2.25 | - | 0.57 | 0.14 | 0.07 | - | 6 | 4 | 3 | - |
| ***CTNNB1*** | snv | c.121A>G | 11.3 | 9.2 | 3.9 | 2.6 | - | 10.6 | 4.3 | 1.8 | 1.7 | 9.9 | 4.1 | 2.2 | 1.7† | 0.99 | 0.28 | 0.57 | - | 10 | 7 | 26 | - |
| ***EGFR*** | deletion | c.2236_2250delGAATTAAGAGAAGCA | 11.3 | 11.3 | 3.6 | 2.5 | - | 12 | 4 | - | - | 11.7 | 3.8 | 2.5† | - | 0.49 | 0.28 | - | - | 4 | 7 | - | - |
| ***EGFR*** | snv | c.2573T>G | 10.9 | 10.8 | 4.6 | 2.3 | - | 10.9 | 4 | 2.2 | 1.4 | 10.9 | 4.3 | 2.25 | 1.4† | 0.07 | 0.42 | 0.07 | - | 1 | 0 | 3 | - |
| ***EGFR,***  ***EGFR-AS1*** | insertion | c.2310_2311insGGT | 9.9 | 11.2 | 3.9 | 1.8 | - | 11.1 | 4.1 | 2.2 | - | 11.2 | 4 | 2 | - | 0.07 | 0.14 | 0.28 | - | 1 | 4 | 4 | - |
| ***EGFR***  ***,EGFR-AS1*** | snv | c.2369C>T | 9.9 | 11.3 | 3.9 | 2.2 | - | 11.4 | 4.4 | 2 | - | 11.4 | 4.15 | 2.1 | - | 0.07 | 0.35 | 0.14 | - | 1 | 9 | 7 | - |
| ***ERBB2*** | insertion | c.2313_2324dupATACGTGATGGC | 8.1 | 7.8 | 2.9 | - | - | 6.8 | 3.3 | - | - | 7.3 | 3.1 | - | - | 0.71 | 0.28 | - | - | 10 | 9 | - | - |
| ***FGFR3*** | snv | c.746C>G | 9.7 | 8.8 | - | - | - | 6.8 | 3 | - | - | 7.8 | 3† | - | - | 1.41 | - | - | - | 18 | - | - | - |
| ***FLT3*** | snv | c.2503G>T | 9.4 | 8.5 | 2.8 | 2.5 | - | 8.3 | 3.8 | 1.9 | - | 8.4 | 3.3 | 2.2 | - | 0.14 | 0.71 | 0.42 | - | 2 | 21 | 19 | - |
| ***GNA11*** | snv | c.626A>T | 11.1 | 11.7 | 4.2 | 3.4 | - | 13.4 | 5.8 | - | - | 12.6 | 5 | 3.4† | - | 1.20 | 1.13 | - | - | 10 | 23 | - | - |
| ***GNAQ*** | snv | c.626A>C | 10.1 | 10.6 | 3.7 | 2.1 | 1.8 | 10.9 | 3.5 | 2.6 | - | 10.8 | 3.6 | 2.35 | 1.8† | 0.21 | 0.14 | 0.35 | - | 2 | 4 | 15 | - |
| ***IDH1*** | snv | c.394C>T | 9.4 | 10.3 | 3.2 | 2.5 | - | 10.5 | 4.7 | 2.6 | - | 10.4 | 3.95 | 2.55 | - | 0.14 | 1.06 | 0.07 | - | 1 | 27 | 3 | - |
| ***JAK2*** | snv | c.1849G>T | 9.1 | 9.5 | 2.9 | 2.2 | - | 9.3 | 3.1 | 1.9 | - | 9.4 | 3 | 2.05 | - | 0.14 | 0.14 | 0.21 | - | 2 | 5 | 10 | - |
| ***KIT*** | snv | c.2447A>T | 9.4 | 10.2 | 4.6 | 2.1 | - | 10 | 3.7 | 2.1 | - | 10.1 | 4.2 | 2.1 | - | 0.14 | 0.64 | 0.00 | - | 1 | 15 | 0 | - |

**Suplementary table 2. Variants detected by different operators at different VAF (Reproducibility and Limit of detection).** SD: standard deviation; CV: coefficient of variation, -: not detected; †: variant found by just one operator.

| ***KRAS*** | snv | c.35G>A | 8.3 | 9.3 | 3.4 | 2.2 | 1.6 | 10.4 | 3.7 | 2.6 | 1.7 | 9.85 | 3.6 | 2.4 | 1.65 | 0.78 | 0.21 | 0.28 | 0.07 | 8 | 6 | 12 | 4 |
| --- | --- | --- | --- | --- | --- | --- | --- | --- | --- | --- | --- | --- | --- | --- | --- | --- | --- | --- | --- | --- | --- | --- | --- |
| ***MPL*** | snv | c.1544G>T | 9.8 | 11.4 | 4.3 | 2.2 | - | 9.6 | 4.2 | 3.6 | - | 10.5 | 4.3 | 2.9 | - | 1.27 | 0.07 | 0.99 | - | 12 | 2 | 34 | - |
| ***NPM1*** | insertion | c.860_863dupTCTG | 8.9 | 8.9 | 2.7 | 1.7 | - | 7.6 | 3.6 | 2.3 | - | 8.25 | 3.15 | 2 | - | 0.92 | 0.64 | 0.42 | - | 11 | 20 | 21 | - |
| ***NRAS*** | snv | c.182A>G | 10 | 9.8 | 5.5 | 3 | - | 10.7 | 4.5 | - | - | 10.3 | 5 | 3† | - | 0.64 | 0.71 | - | - | 6 | 14 | - | - |
| ***PDGFRA*** | insertion | c.1694_1695insA | 9 | 12.8 | 3.2 | 3.4 | - | 11.8 | 6.2 | 2.8 | - | 12.3 | 4.7 | 3.1 | - | 0.71 | 2.12 | 0.42 | - | 6 | 45 | 14 | - |
| ***PDGFRA*** | snv | c.2525A>T | 10.7 | 8.6 | 4.4 | 2.6 | - | 9.5 | 3.8 | 2 | 1.6 | 9.05 | 4.1 | 2.3 | 1.6† | 0.64 | 0.42 | 0.42 | - | 7 | 10 | 18 | - |
| ***PIK3CA*** | snv | c.1633G>A | 9.2 | 11 | 4.4 | 2.4 | - | 10.9 | 4.1 | 2.2 | 1.4 | 11 | 4.25 | 2.3 | 1.4† | 0.07 | 0.21 | 0.14 | - | 1 | 5 | 6 | - |
| ***PIK3CA*** | snv | c.3140A>G | 8.8 | 9.5 | 3.4 | 1.9 | - | 10.1 | 3.8 | 2.1 | - | 9.8 | 3.6 | 2 | - | 0.42 | 0.28 | 0.14 | - | 4 | 8 | 7 | - |
| ***PIK3CA*** | insertion | c.3204_3205insA | 8.9 | 9.5 | 3.6 | 2 | - | 9.5 | 4.5 | 2.2 | 1.6 | 9.5 | 4.05 | 2.1 | 1.6† | 0.00 | 0.64 | 0.14 | - | 0 | 16 | 7 | - |
| ***PTEN*** | insertion | c.741dupA | 9.8 | 12.4 | 4.8 | 2.2 | - | 10.4 | 4.2 | 1.8 | - | 11.4 | 4.5 | 2 | - | 1.41 | 0.42 | 0.28 | - | 12 | 9 | 14 | - |
| ***PTEN*** | deletion | c.800delA | 9.8 | 12.7 | 5.4 | 2.3 | 1.7 | 10.7 | 4.7 | 2 | - | 11.7 | 5.05 | 2.15 | 1.7† | 1.41 | 0.49 | 0.21 | - | 12 | 10 | 10 | - |
| ***RET*** | snv | c.2753T>C | 9.1 | 10.8 | 4.6 | 2 | 1.6 | 10.9 | 4.6 | 2.7 | - | 10.9 | 4.6 | 2.35 | 1.6† | 0.07 | 0.00 | 0.49 | - | 1 | 0 | 21 | - |
| ***TP53*** | snv | c.818G>A | 9 | 10.1 | 3 | 2.6 | - | 11.1 | 3.1 | 2.9 | - | 10.6 | 3.05 | 2.75 | - | 0.71 | 0.07 | 0.21 | - | 7 | 2 | 8 | - |
| ***TP53*** | snv | c.743G>A | 9 | 7.3 | 2.8 | - | - | 7.8 | 2.8 | - | - | 7.55 | 2.8 | - | - | 0.35 | 0.00 | - | - | 5 | 0 | - | - |
| ***TP53*** | deletion | c.723delC | 8.7 | 10.5 | 4.2 | 1.8 | - | 10.9 | 4.2 | 2.1 | 2.1 | 10.7 | 4.2 | 1.95 | 2.1† | 0.28 | 0.00 | 0.21 | - | 3 | 0 | 11 | - |
| ***TP53*** | snv | c.524G>A | 9.2 | 10.6 | 4.5 | 2.5 | - | 12.5 | 3.7 | 2.1 | - | 11.6 | 4.1 | 2.3 | - | 1.34 | 0.57 | 0.28 | - | 12 | 14 | 12 | - |
| ***TP53*** | deletion | c.267delC | NA | 10.7 | 4.1 | 2.6 | - | 12.8 | 3.3 | 2.2 | 2 | 11.8 | 3.7 | 2.4 | 2† | 1.48 | 0.57 | 0.28 | - | 13 | 15 | 12 | - |

**Supplementary Table 3.** Variant distribution and clinical impact.

| Patient | Gene | Mutation | VAF | Prognostic | Therapeutic | POTENTIAL GERMLINE | ref |
| --- | --- | --- | --- | --- | --- | --- | --- |
| 1 | *NRAS* | p.Gly13Arg; c.37G>C | 12,5 | x | - | - | ^1–3^ |
|  | *KRAS* | p.Gly13Val; c.38G>T | 22 | x | - | - | ^1–3^ |
| 2 | *NRAS* | p.Gly12Cys; c.34G>T | 14,8 | x | - | - | ^1–3^ |
|  | *KRAS* | p.Leu19Phe; c.57G>C | 5,6 | x | - | - | ^1–3^ |
| 3 | *NRAS* | p.Gly12Cys; c.34G>T | 5 | x | - | - | ^1–3^ |
|  | *FLT3* | p.Tyr842Cys;c.2525A>G | 46 | x | x | - | ^4–6^ |
|  | *PTPN11* | p.Ala72Asp; c.215C>A | 5 | - | - | - | **-** |
| 4 | *NRAS* | p.Gly60Glu; c.179G>A | 16 | x | - | - | ^1–3^ |
|  | *PTPN11* | p.Ala72Thr; c.214G>A | 3,2 | - | - | - | **-** |
| 5 | *NRAS* | p.Gln61Lys; c.181C>A | 48,5 | x | - | - | ^1–3^ |
|  | *RUNX1* | p.Ile366_Gly367dup; c.1098_1103dupCGGCAT | 48,7 | x | x | x | ^7,8^ |
| 6 | *NRAS* | p.Gly12Asp; c.35G>A | 44,7 | x |  | - | ^1–3^ |
|  | *TP53* | p.Val274Leu; c.820G>C | 76,7 | x | x | x | ^9–11^ |
| 7 | *NRAS* | p.Gly13Cys;c.37G>T | 6,7 | x | - | - | ^1–3^ |
|  | *BRAF* | p.Asp594Asn;c.1780G>A | 5,9 | - | - | - | **-** |
|  | *MTOR* | p.Val2406Met; c.7216G>A | 7,3 | - | x | - | ^12^ |
|  | *DDX3X* | p.Tyr466His;c.1396T>C | 52,5 | - | - | - | **-** |
| 8 | *NRAS* | p.Gly13Arg; c.37G>C | 14,2 | x | - | - | ^1–3^ |
| 9 | *NRAS* | p.Gly13Asp; c.38G>A | 7,5 | x | - | - | ^1–3^ |
| 10 | *NRAS* | p.Gly12Ser; c.34G>A | 39 | x | - | - | ^1–3^ |
| 11 | *NRAS* | p.Gln61Arg;c.181_182delCAinsAG | 30,4 | x | - | - | ^1–3^ |
| 12 | *FLT3* | p.Gly12Asp; c.35G>A | 5 | x | x | - | ^4–6^ |
|  | *PTPN11* | p.Val592Ala; c.1775T>C | 13,4 | - | - | - | **-** |
| 13 | *KRAS* | p.Gly12Asp; c.35G>C | 5 | x | - | - | ^1–3^ |
|  | *FLT3* | p.Asp593Gly;c.1778A>G | 5 | x | x | - | ^4–6^ |
| 14 | *KRAS* | p.Gly12Asp; c.35G>A | 5 | x | - | - | ^1–3^ |
|  | *FLT3* | p.Ile836del; c.2508_2510delCAT | 5 | x | - | - | ^1–3^ |
| 15 | *KRAS* | p.Gly12Val; c.35G>T | 44 | x | - | - | ^1–3^ |
|  | *PAX5* | p.Ile181Hisfs*62; c.540dupC | 25 | x | - | - | ^1–3^ |
| 16 | *KRAS* | p.Leu23Arg; c.68T>G | 30,5 | x | - | - | ^1–3^ |
| 17 | *KRAS* | p.Gly12Val;c.35G>T | 21 | x | - | - | ^1–3^ |
| 18 | *KRAS* | p.Val14Ile;c.40G>A | 45,9 | x | - | - | ^1–3^ |
| 19 | *FLT3* | p.Ile836del;c.2508_2510delCAT | 30 | x | x | - | ^4–6^ |
|  | *PTPN11* | p.Glu76Asp; c.228G>T | 12 | - | - | - | - |
| 20 | *NRAS* | p.Gly13Asp; c.38G>A | 2,6 | x | - | - | ^1–3^ |
|  | *KRAS* | p.Gly12Ala; c.35G>C | 27,1 | x | - | - | ^1–3^ |
| 21 | *PTPN11* | p.Glu69Lys;c.205G>A | 30,2 | - | - | - | - |
|  | *TP53* | p.Arg248Gln; c.743G>A | 5 | x | x | - | ^9–11^ |
|  | *MSH6* | p.Gln1334del; c.3999_4001delGCA | 5 | - | - | x |  |
|  | *ASXL1* | p.Ser577*; c.1730C>G | 30 | x | x | - | ^13,14^ |
|  | *NT5C2* | p.His352Asp; c.1054C>G | 13 | - | x | - | ^15–17^ |
| 22 | *FLT3* | p.Gly846Ser; c.2536G>A | 34 | x | x |  | ^4–6^ |
| 23 | *MSH6* | p.Phe1088SerfsTer2; c.3261delC | 2,1 | - | - | x | ^18^ |
|  | *ASXL1* | p.Gly643fs; c.1927delG | 2,1 | x | x |  | ^13,14^ |
| 24 | *TPMT* | p.Ala80Pro; c.238G>C | 50,7 | - | x | x | ^19–23^ |
|  | *ASXL1* | p.Gly643fs; c.1927delG | 2,5 | x | x |  | ^13,14^ |
|  | *PRPS1* | p.Ala105Gly; c.314C>G | 42 | x | x | - | ^13,14^ |
| 25 | *RUNX1* | p.Arg107His; c.320G>A | 31,6 | x | x | x | ^7,8^ |
|  | *TPMT* | p.Ala80Pro; c.238G>C | 47,6 | - | x | x | ^19–23^ |
| 26 | *PAX5* | p.Pro80Arg; c.239C>G | 8,1 | x | - | - | ^24,25^ |
|  | *TP53* | p.Arg290His; c.869G>A | 50,6 | x | x | - | ^9–11^ |
| 27 | *ASXL2* | p.Arg614*;c.1840C>T | 33,5 | - | - | - | - |
|  | *MSH6* | p.Arg1095Cys;c.3283C>T | 48 | - | - | x | ^18^ |
| 28 | *ASXL2* | p.Thr740Lys;c.2219C>A | 12 | - | - |  | - |
| 29 | *PAX5* | p.Ala322Argfs*19, c.963dupC | 35 | x | - | - | ^24,25^ |
| 30 | *IKZF1* | p.Ser193TrpfsTer?;c.575_576insGGGA | 40,8 | x | x | x | ^26–28^ |
| 31 | *IKZF1* | p.Gln95Ter;c.281C>T | 47 | x | x | x | ^26–28^ |
| 32 | *JAK1* | p.Arg724Cys;c.217C>T | 50,3 | x | x | - | ^29–31^ |
| 33 | *KMTD2* | p.Gln3745*;c.11233C>T | 31,3 | - | - | - | - |
| 34 | *SMARCB1* | p.Gly305Arg;c.913G>C | 51,4 | -- | - | - | - |
| 52 | *NRAS* | p.Ala146Thr; c.436G>A | 41,3 | x | - | - | ^1–3^ |
| 53 | *NOTCH1* | p.Gln2501Ter; c.7501C>T | 9.9 | x | x | - | ^32–34^ |
|  | *PTEN* | p.Ile253AsnfsTer45; c.757dupA | 37.4 | x | x | - | ^32–34^ |
|  | *STAT5B* | STAT5B p.Asn642His;c.1924A>C |  | - | - | - | - |
| 54 | *ATRX* | p.Lys1103*; c.3307A>T | 54.7 | - | - |  | - |
|  | *JAK3* | p.Arg657Gln; c.1970G>A | 47 | x | x | - | ^29–31^ |
|  | *FBXW7* | p.Arg465His; c.1394G>A | 56.7 | x | x | - | ^32–34^ |
| 55 | *NOTCH1* | p.Leu1678Pro; c.5033T>C | 45.3 | x | x | - | ^32–34^ |
|  | *TPMT* | p.Ala80Pro; c.238G>C | 57.1 | - | x | x | ^19–23^ |
| 56 | *NOTCH1* | p.Val1676Asp: c.5027T>A  +  p.His1601Pro; c.4802A>C | 5 | x | x | - | ^32–34^ |
| 57 | *NOTCH1* | p.Val1605dup; c.4809_4811dupCGT | 30.3 | x | x | - | ^32–34^ |
| 58 | *NOTCH1* | p.Val2453CysfsTer24; c.7357delG  +  p.Ala382ProfsTer7; c.1142_1143insCCCCCGTAGGATC | 44 | x | x | - | ^32–34^ |
| 59 | *RUNX1* | p.Ser94Arg;c.282C>G | 50 | x | x | x | ^7,8^ |
| 60 | *NF1* | p.Thr2507Ile; c.7520C>T | 45.7 |  |  |  |  |
|  | *PTEN* | p.Leu247Cysfs*10; c.3127_3129delAGC | 37.4 | x | x | - | ^32–34^ |
| 61 | *PTEN* | p.Leu70Phe; c.208C>T + p.Leu247Glyfs*12; c.738_739insGGTCCGTG | 46.7 | x | x | - | ^32–34^ |
| 62 | *NRAS* | p.Gly13Asp; c.38G>A | 38.4 | x | - | - | ^1–3^ |
|  | *ASXL2* | p.Arg741Ter; c.2219_2220insCT  +  p.Thr740Lys; c.2219C>A | 38.4 | - | - |  | - |
| 63 | *PTPN11* | p.Thr507Lys; c.1520C>A | 21.1 | - | - | - | - |
|  | *IDH1* | p.Arg132His; c.395G>A | 23 | x | x | - | ^35–37^ |
| 64 | *PTPN11* | p.Asp61His  c.181G>C | 49 | - | - | - | - |
| 65 | *FLT3* | p.Tyr597_Glu598insAspTyrValAspPheArgGluTyr; c.1770_1793dupCTACGTTGATTTCAGAGAATATGA | 38 | x | x | **-** | ^4–6^ |
|  | *IDH2* | p.Arg140Gln; c.419G>A | 49 | x | x | - | ^35–37^ |
| 66 | *NPM1* | p.Trp288CysfsTer12; c.863_864insTGTA | 47.3 | x | x | - | ^38,39^ |
|  | *WT1* | p.Ala382TyrfsTer70; c.1159_1161insTACGGTCG | 86,1 | - | - | - | - |
| 67 | *RUNX1* | p.Gly217Glu; c.650G>A | 49 | x | x | x | ^7,8^ |
| 68 | *MET* | p.Thr1010Ile; c.3029C>T | 20.1 | - | - |  | - |
| 69 | *APC* | p.Leu563ValfsTer18; c.1685_1686insA | 55 | - | - |  | - |

**Supplementary table 4.** Cytogenetic information from patients lacking any alteration after performing the AmpliSeq^TM^ for Illumina® Childhood Cancer Panel

| **Patient** | **Sex** | **Leukemia subtype** | **Kariotype** |
| --- | --- | --- | --- |
| Patient 1 | Female | B-ALL | 47,XX,+X,-21,+mar.ish der(21)(RUNX1 amp) [15]/46,XX[10] |
| Patient 2 | Female | B-ALL | Not available |
| Patient 3 | Female | B-ALL | Not available |
| Patient 4 | Female | B-ALL | 57,XX,+X,+4,+6,+7,+8,+10,+14,+17,+18,+21,+21[13]/46,XX[7] |
| Patient 5 | Female | B-ALL | 46,XX,del(16)(q10)[4]/46,XX,der(16)t(1;16)(q?25;q?22)[19]/46,XX[5] |
| Patient 6 | Female | B-ALL | 47,XX,der(2),+5,del(6q)[7]/47,XX, der(2),+del(5q),del(6q)[7]/48,XX, der(2),der(2),+der(2),+del(5q),del(6q)[4]/46,XX[1] |
| Patient 7 | Male | B-ALL | 46 XY |
| Patient 8 | Female | B-ALL | 46,XX[20] |
| Patient 9 | Female | B-ALL | 46,XX,t(11;14)(q23;q32)[15]/47,XX,idem,+mar[5]/46,XX[13] |
| Patient 10 | Male | T-ALL | Not available |
| Patient 11 | Male | LMA | 47,XY,del(9)(p13),-14,-20,+21,+21c,+mar[11]/47,XY,+21c[48] |
| Patient 12 | Female | LMA | 46,XX,der(17)(p),del(19)[14] |
| Patient 13 | Male | LMA | 44,XY,+der(1)t(1;?)(?p13;?),-9,-10,der(17)t(17;?)(?p11:?), -18[13]/46,XY[17] |

**References**

1. Ariës IM, van den Dungen RE, Koudijs MJ, et al. Towards personalized therapy in pediatric acute lymphoblastic leukemia: RAS mutations and prednisolone resistance. *Haematologica*. 2015;100(4):e132–e136.

2. Jerchel IS, Hoogkamer AQ, Ariës IM, et al. RAS pathway mutations as a predictive biomarker for treatment adaptation in pediatric B-cell precursor acute lymphoblastic leukemia. *Leukemia*. 2018;32(4):931–940.

3. Case M, Matheson E, Minto L, et al. Mutation of genes affecting the RAS pathway is common in childhood acute lymphoblastic leukemia. *Cancer Res.* 2008;68(16):6803–9.

4. Conneely SE, Rau RE. The genomics of acute myeloid leukemia in children. *Cancer Metastasis Rev.* 2020;39(1):189–209.

5. Daver N, Schlenk RF, Russell NH, Levis MJ. Targeting FLT3 mutations in AML: review of current knowledge and evidence. *Leukemia*. 2019;33(2):299–312.

6. Schwartz GW, Manning B, Zhou Y, et al. Classes of ITD predict outcomes in AML patients treated with FLT3 inhibitors. *Clin. Cancer Res.* 2019;25(2):572–583.

7. Yokota A, Huo L, Lan F, Wu J, Huang G. The Clinical, Molecular, and Mechanistic Basis of RUNX1 Mutations Identified in Hematological Malignancies. *Mol. Cells*. 2020;

8. Sood R, Kamikubo Y, Liu P. Role of RUNX1 in hematological malignancies. *Blood*. 2017;129(15):2070–2082.

9. Forero-Castro M, Robledo C, Benito R, et al. Mutations in TP53 and JAK2 are independent prognostic biomarkers in B-cell precursor acute lymphoblastic leukaemia. *Br. J. Cancer*. 2017;117(2):256–265.

10. Comeaux EQ, Mullighan CG. TP53 mutations in hypodiploid acute lymphoblastic leukemia. *Cold Spring Harb. Perspect. Med.* 2017;7(3):.

11. Demir S, Boldrin E, Sun Q, et al. Therapeutic targeting of mutant p53 in pediatric acute lymphoblastic leukemia. *Haematologica*. 2019;haematol.2018.199364.

12. Simioni C, Martelli A, Zauli G, Melloni E, Neri L. Targeting mTOR in Acute Lymphoblastic Leukemia. *Cells*. 2019;8(2):190.

13. L B, K D, H D. Genomics of Acute Myeloid Leukemia Diagnosis and Pathways. *J. Clin. Oncol.* 2017;35(9):.

14. Al-harbi S, Aljurf M, Mohty M, et al. An update on the molecular pathogenesis and potential therapeutic targeting of AML with t ( 8 ; 21 )( q22 ; q22 . 1 ); RUNX1-RUNX1T1 Molecular pathogenesis of. 2020;4(1):.

15. Dieck CL, Ferrando A. Genetics and mechanisms of NT5C2-driven chemotherapy resistance in relapsed ALL. *Blood*. 2019;133(21):2263–2268.

16. Tzoneva G, Dieck CL, Oshima K, et al. Clonal evolution mechanisms in NT5C2 mutant-relapsed acute lymphoblastic leukaemia. *Nature*. 2018;553(7689):511–514.

17. Barz MJ, Hof J, Groeneveld-Krentz S, et al. Subclonal NT5C2 mutations are associated with poor outcomes after relapse of pediatric acute lymphoblastic leukemia. *Blood*. 2020;

18. Evensen NA, Madhusoodhan PP, Meyer J, et al. MSH6 haploinsufficiency at relapse contributes to the development of thiopurine resistance in pediatric B-lymphoblastic leukemia. *Haematologica*. 2018;103(5):830–839.

19. McLeod HL, Krynetski EY, Relling M V., Evans WE. Genetic polymorphism of thiopurine methyltransferase and its clinical relevance for childhood acute lymphoblastic leukemia. *Leukemia*. 2000;14(4):567–572.

20. Mei L, Ontiveros EP, Griffiths EA, et al. Pharmacogenetics predictive of response and toxicity in acute lymphoblastic leukemia therapy. *Blood Rev.* 2015;29(4):243–249.

21. Azimi F, Jafariyan M, Khatami S, Mortazavi Y, Azad M. Assessment of Thiopurine-based drugs according to Thiopurine S-methyltransferase genotype in patients with Acute Lymphoblastic Leukemia. *Iran. J. Pediatr. Hematol. Oncol.* 2014;4(1):32–8.

22. Stanulla M, Schaeffeler E, Flohr T, et al. Thiopurine methyltransferase (TPMT) genotype and early treatment response to mercaptopurine in childhood acute lymphoblastic leukemia. *JAMA*. 2005;293(12):1485–9.

23. Lennard L, Cartwright CS, Wade R, Vora A. Thiopurine dose intensity and treatment outcome in childhood lymphoblastic leukaemia: The influence of thiopurine methyltransferase pharmacogenetics. *Br. J. Haematol.* 2015;169(2):228–240.

24. Gu Z, Churchman ML, Roberts KG, et al. PAX5-driven subtypes of B-progenitor acute lymphoblastic leukemia. *Nat. Genet.* 2019;51(2):296–307.

25. Schwab C, Harrison CJ. Advances in B-cell Precursor Acute Lymphoblastic Leukemia Genomics. *HemaSphere*. 2018;2(4):e53.

26. Stanulla M, Cavé H, Moorman A V. IKZF1 deletions in pediatric acute lymphoblastic leukemia: still a poor prognostic marker? *Blood*. 2020;135(4):252–260.

27. Stanulla M, Dagdan E, Zaliova M, et al. IKZF1plus Defines a New Minimal Residual Disease-Dependent Very-Poor Prognostic Profile in Pediatric B-Cell Precursor Acute Lymphoblastic Leukemia. *J. Clin. Oncol.* 2018;36(12):1240–1249.

28. Marke R, van Leeuwen FN, Scheijen B. The many faces of IKZF1 in B-cell precursor acute lymphoblastic leukemia. *Haematologica*. 2018;103(4):565–574.

29. Roberts KG, Mullighan CG. Genomics in acute lymphoblastic leukaemia: insights and treatment implications. *Nat. Rev. Clin. Oncol.* 2015;

30. Zhang J, Ding L, Holmfeldt L, et al. The genetic basis of early T-cell precursor acute lymphoblastic leukaemia. *Nature*. 2012;481(7380):157–163.

31. Girardi T, Vicente C, Cools J, De Keersmaecker K. The genetics and molecular biology of T-ALL. *Blood*. 2017;129(9):1113–1123.

32. Jenkinson S, Koo K, Mansour MR, et al. Impact of NOTCH1/FBXW7 mutations on outcome in pediatric T-cell acute lymphoblastic leukemia patients treated on the MRC UKALL 2003 trial. *Leukemia*. 2013;27(1):41–47.

33. Petit A, Trinquand A, Chevret S, et al. Oncogenetic mutations combined with MRD improve outcome prediction in pediatric T-cell acute lymphoblastic leukemia. *Blood*. 2018;131(3):289–300.

34. N V-G, S P-J, E E-C, et al. Measurable Residual Disease Assessed by Flow-Cytometry Is a Stable Prognostic Factor for Pediatric T-Cell Acute Lymphoblastic Leukemia in Consecutive SEHOP Protocols Whereas the Impact of Oncogenetics Depends on Treatment. *Front. Pediatr.* 2021;8:.

35. Nassereddine S, Lap CJ, Haroun F, Tabbara I. The role of mutant IDH1 and IDH2 inhibitors in the treatment of acute myeloid leukemia. *Ann. Hematol.* 2017;96(12):1983–1991.

36. Creutzig U, van den Heuvel-Eibrink MM, Gibson B, et al. Diagnosis and management of acute myeloid leukemia in children and adolescents: recommendations from an international expert panel. *Blood*. 2012;120(16):3187–205.

37. AK A, DW M, JA L, et al. IDH1 and IDH2 Mutations in Pediatric Acute Leukemia. *Leukemia*. 2011;25(10):.

38. Heath EM, Chan SM, Minden MD, et al. Biological and clinical consequences of NPM1 mutations in AML. *Leukemia*. 2017;31(4):798–807.

39. Bolouri H, Farrar JE, Triche T, et al. The molecular landscape of pediatric acute myeloid leukemia reveals recurrent structural alterations and age-specific mutational interactions. *Nat. Med.* 2017;24(1):103–112.
